# Supplementary material for: Predictors of youth unemployment duration and impact evaluation of job creation program in East Gojjam Zone
Source: PLoS One. 2025 Apr 4;20(4):e0320795. doi: 10.1371/journal.pone.0320795 (PMC11970665; doi:10.1371/journal.pone.0320795)
Supplement: S1 Table — Results of Concordance Measures, Model Fit, and Schoenfeld Residuals Test (DOCX) [file pone.0320795.s001.docx]

S1Table: Results of Concordance Measures, Model Fit, and Schoenfeld Residuals Test

| Category | Values |
| --- | --- |
| Number of Subjects | 240 |
| Number of Comparison Pairs | 12148 |
| Number of Expected Orderings | 9198 |
| Number of Tied Predictions | 66 |
| Harrell's C | 0.7700 |
| Somers' D | 0.5400 |
| Asymptotic SE | 0.01892 |
| Log-likelihood of the null model | -389.0274 |
| Log-likelihood of the Model | -352.3814 |
| AIC | 742.7629 |
| BIC | 808.895 |
| Schoenfeld Residuals Chi-Square Test | 7.33 |
| Degrees of Freedom | 13 |
| P-value | 0.8841 |
