# Supplementary material for: Predictors of youth unemployment duration and impact evaluation of job creation program in East Gojjam Zone
Source: PLoS One. 2025 Apr 4;20(4):e0320795. doi: 10.1371/journal.pone.0320795 (PMC11970665; doi:10.1371/journal.pone.0320795)
Supplement: S2 Table — Results of multiple logistics regression on the participation of job creation programs in East Gojjam Zone, North West Ethiopia (DOCX) [file pone.0320795.s002.docx]

S2 Table: Results of multiple logistics regression on the participation of job creation programs in East Gojjam Zone, North West Ethiopia

| Variables | Category | Coef | S.E. | P-value | AOR |
| --- | --- | --- | --- | --- | --- |
| Sex | Female | -2.277 | 0.517 | <0.001 | 0.103 |
|  | male (ref) |  |  |  |  |
| Age | Continuous | 0.337 | 0.11 | 0.002 | 1.4 |
| Education level | Certificate or below | 6.123 | 2.1 | 0.004 | 456.179 |
|  | Diploma | -0.768 | 0.506 | 0.129 | 0.464 |
|  | Degree (reference) |  |  |  |  |
| Business consultant services | No | -2.729 | 0.838 | 0.001 | 0.065 |
|  | Yes (ref) |  |  |  |  |
| Experience in participation of job creation programs | No | -1.405 | 0.476 | 0.003 | 0.245 |
|  | Yes (ref) |  |  |  |  |
| Mother's job | Public employment | 1.114 | 1.246 | 0.372 | 3.045 |
|  | Run their business | 4.889 | 1.485 | 0.001 | 132.842 |
|  | Others (ref) |  |  |  |  |
| Field of the study | Agriculture | 5.466 | 2.362 | 0.021 | 236.468 |
|  | Business, Economics, or Social Science | 5.423 | 2.155 | 0.012 | 226.586 |
|  | Engineering | 6.411 | 2.168 | 0.003 | 608.28 |
|  | Other (reference) |  |  |  |  |
| Job preference | Public employment | 0.037 | 0.463 | 0.936 | 1.038 |
|  | Non-government employment | 1.359 | 0.749 | 0.07 | 3.892 |
|  | Ownership or partnerships (ref) |  |  |  |  |
| Woreda | Debre Markos | 2.891 | 0.843 | 0.001 | 18.019 |
|  | Debre Elias | -4.854 | 1.241 | <0.001 | 0.008 |
|  | Sinan | -4.114 | 1.101 | <0.001 | 0.016 |
|  | Debre Wereke | 0.737 | 0.712 | 0.301 | 2.089 |
|  | Bichena | 1.818 | 0.742 | 0.014 | 6.157 |
|  | Awabel (ref) |  |  |  |  |
| Constant | Constant | -10.734 | 3.694 | 0.004 | 0.000 |

Coef=regression coefficient, S.E=standard error, P-value=probability value, AOR=Adjusted odd ratio, ref=reference category
