# Supplementary material for: Predictors of youth unemployment duration and impact evaluation of job creation program in East Gojjam Zone
Source: PLoS One. 2025 Apr 4;20(4):e0320795. doi: 10.1371/journal.pone.0320795 (PMC11970665; doi:10.1371/journal.pone.0320795)
Supplement: S3 Table — Results of multiple linear regression analysis on the predictors of earnings (DOCX) [file pone.0320795.s003.docx]

S3 Table: Results of multiple linear regression analysis on the predictors of earnings

| Variables | Category | Coef | S. E | P-value |
| --- | --- | --- | --- | --- |
| Duration of unemployment | Continuous | -51.211 | 15.511 | 0.001 |
| Experience in participation of job creation program | No | -1417.521 | 524.490 | 0.007 |
|  | (Ref) |  |  |  |
| Adequate job information | No | -1922.049 | 524.925 | <0.001 |
|  | (Ref) |  |  |  |
| Father's job | Government employee | 2131.550 | 915.886 | 0.021 |
|  | run their own business | -1669.143 | 895.241 | 0.064 |
| Field of the study | Business Economics or Social Science | -350.384 | 1218.846 | 0.774 |
|  | Engineering | -1636.597 | 866.160 | 0.060 |
|  | (Ref) | -867.710 | 766.536 | 0.259 |
| Participation in job creation | Yes | 1534.982 | 498.972 | 0.002 |
|  | (Ref) |  |  |  |
| Constants |  | 6138.340 | 847.368 | <0.001 |

Coef=regression coefficient, S.E=standard error, P-value=probability value, ref=reference category
