# Supplementary material for: Predictors of youth unemployment duration and impact evaluation of job creation program in East Gojjam Zone
Source: PLoS One. 2025 Apr 4;20(4):e0320795. doi: 10.1371/journal.pone.0320795 (PMC11970665; doi:10.1371/journal.pone.0320795)
Supplement: S4 Table — Results of Hosmer-Lemeshow Test (DOCX) [file pone.0320795.s004.docx]

S4 Table: Results of Hosmer-Lemeshow Test

| Statistic | Multiple Logistic Regression Model | Propensity Score Model |
| --- | --- | --- |
| Hosmer-Lemeshow test statistic | 5.22 | 3.29 |
| P-value | 0.7338 | 0.9149 |

P-value=probability value
