# Supplementary material for: Predictors of youth unemployment duration and impact evaluation of job creation program in East Gojjam Zone
Source: PLoS One. 2025 Apr 4;20(4):e0320795. doi: 10.1371/journal.pone.0320795 (PMC11970665; doi:10.1371/journal.pone.0320795)
Supplement: S5 Table — Covariate Balance Assessment (DOCX) [file pone.0320795.s005.docx]

S5 Table: Covariate Balance Assessment

| **Variables** | **categories** | Standardized mean differences | | Variance ratio | |
| --- | --- | --- | --- | --- | --- |
|  |  | Raw | Matched | Raw | Matched |
| Sex | Female | -0.294 | -0.015 | 1.216 | 1.015 |
|  | male(ref) |  |  |  |  |
| Age | continuous | 0.272 | 0.006 | 1.326 | 1.033 |
| Education level | certificate or below | 0.637 | 0.032 | 22.168 | 1.091 |
|  | diploma | -0.217 | -0.032 | 0.806 | 1.027 |
|  | degree(ref) |  |  |  |  |
| Field of the study | Agriculture | -0.283 | -0.018 | 0.868 | 1.060 |
|  | Business Economics or Social Science | -0.131 | 0.048 | 0.852 | 1.045 |
|  | Engineering | 0.184 | -0.035 | 0.930 | 1.012 |
|  | others(ref) |  |  |  |  |
| Woreda | Debre Markos | 0.330 | -0.007 | 2.263 | 0.919 |
|  | Debre Elias | -0.330 | -0.067 | 1.423 | 0.917 |
|  | Sinan | -0.422 | -0.012 | 0.455 | 0.888 |
|  | Diver Wereke | 0.008 | 0.057 | 1.015 | 1.069 |
|  | Bichena | 0.644 | 0.025 | 2.919 | 1.073 |
|  | Awabel(ref) |  |  |  |  |
| Length of time to job seeker in years | continuous | 0.254 | 0.006 | 2.224 | 0.935 |
| Business consultant services | no | -0.358 | -0.0019 | 1.301 | 0.963 |
|  | yes(ref) |  |  |  |  |
| Job Selection | Public Employment | -0.469 | -0.0016 | 0.983 | 0.975 |
|  | Non-Governmental Organization | 0.088 | 0.002 | 1.251 | 1.054 |
|  | partnership or ownerships(ref) |  |  |  |  |
| Experience of participation in job creation | no | -0.376 | -0.0018 | 0.908 | 0.997 |
|  | yes(ref) |  |  |  |  |
| Father's job | Government employee | 0.427 | 0.000 | 4.629 | 1.000 |
|  | run their own business | 0.116 | -0.050 | 1.455 | 0.911 |
|  | others(ref) |  |  |  |  |
| Mother's job | Government employee | 0.305 | -0.009 | 8.526 | 0.910 |
|  | run their own business | 0.305 | -0.051 | 8.526 | 1.072 |
|  | others(ref) |  |  |  |  |
| Adequate job information | no | 0.170 | -0.025 | 1.142 | 0.911 |
|  | yes(ref) |  |  |  |  |
|  | | Raw | | Matched | |
| Number of observation | | 240,000 | | 480,000 | |
| Treated observation | | 96,000 | | 240,000 | |
| Control observation | | 144,000 | | 240,000 | |
