# Supplementary material for: Predictors of youth unemployment duration and impact evaluation of job creation program in East Gojjam Zone
Source: PLoS One. 2025 Apr 4;20(4):e0320795. doi: 10.1371/journal.pone.0320795 (PMC11970665; doi:10.1371/journal.pone.0320795)
Supplement: Attachment — Submitted filename: Review_PONE-D-24-20085R3_en.pdf [file pone.0320795.s010.pdf]

Review

Predictors of youth unemployment duration and impact evaluation of job creation program in East Gojjam Zone

Manuscript Number: PONE-D-24-20085R3

Corresponding Author: Nigusie Gashaye Shita, M.Sc.

The study focuses on the factors that are essential in a given region to help job creation programmes have an impact. The methodology used is fully adapted to the research questions identified. Their results are relevant based on the research methods and their analysis and will help the region to address the problems effectively and strengthen the support system.

The research highlights the problem of youth unemployment in parts of Ethiopia. Their research also highlights job creation initiatives, skills gaps, regional disparities and limited access to food. The study not only articulates the causes but also proposes solutions to achieve this goal. They identify all areas that can help and support inclusive growth through job creation based on their research.

The researchers have revised their study accordingly, with improvements made to the research methodology, stylistic factors and structure. These revisions made the study fully fit for publication.
