# Supplementary material for: Predictors of youth unemployment duration and impact evaluation of job creation program in East Gojjam Zone
Source: PLoS One. 2025 Apr 4;20(4):e0320795. doi: 10.1371/journal.pone.0320795 (PMC11970665; doi:10.1371/journal.pone.0320795)
Supplement: Attachment — Submitted filename: renamed_cc131.docx [file pone.0320795.s006.docx]

Authors’ responses to the comments and suggestions from reviewers on the manuscript “Predictors of Youth Unemployment Duration and Impact Evaluation of Job Creation Program in East Gojjam Zone”

**Authors:**  Nigusie Gashaye Shita ([nigusie27@gmail.com](mailto:nigusie27@gmail.com))

Metadel Azeze Mekonnen^1^: mekonmeti@gmail.com

Yeshiwas Ewinetu Tegegne^2^: yeshiwas@gmail.com

Misganaw Mekonnen Nigussie^1^: misganaa2004@yahoo.com

Awoke Fetahi Woudneh^1^: [fetahi.aw@gmail.com](mailto:fetahi.aw@gmail.com)

**Version:** 1 **Date:** August 6, 2024

**Author’s response to Editorial Board Member and reviews:**

Dear **Editorial Board Member and reviews**,

Thank you very much for your comments and suggestions to improve our manuscript.

Responses to some of the comments are addressed below and the rest of the comments and suggestions are incorporated in the manuscript. Kindly note that changes are highlighted in yellow in the main manuscript.

**Reply to Reviewer 1,**

1. Factors associated with unemployment waiting time were found using the Cox regression. I find this approach very interesting. However, there is no quality assessment of the created regression model. Did you check its predictions using a part of your data as a training sample? Is it possible to list some evaluation statistics for this model?

**Response:** The Cox regression model underwent rigorous evaluation to assess its robustness and predictive accuracy. To ensure model reliability, we assessed the proportional hazards assumption, which was confirmed through analysis of Schoenfeld residuals. Additionally, we evaluated the model's predictive performance using Harrell's C and Gonen-Heller's K, which yielded moderate to high concordance values. Model complexity was assessed using AIC and BIC, indicating an optimal balance between model fit and parsimony. The combination of these evaluation methods provides strong evidence of the model's predictive ability. Further details are available in S1Table .

2. The p-value of Family prosperity – poor is probably incorrect (p-value of 6.758).

**Response:** We have corrected the error in the reported p-value for "Family prosperity – poor." The accurate p-value is 0.043. Thank you for bringing this to our attention.

3. How did you handle the insignificant variables in the Cox regression model? Is their statistical significance important?

**Response:** To identify potential predictors, we employed a stepwise variable selection process. Initially, bivariate analysis was conducted with a significance threshold of 0.2. This was followed by multivariate analysis with a stricter threshold of 0.1 to account for potential confounders. Insignificant variables and their interactions were carefully evaluated during the development of the Cox regression model. Although these variables were initially included, their incorporation did not substantially enhance model performance, as indicated by changes in model fit statistics and the significance of other variables. Model optimization was achieved using likelihood ratio tests, as well as AIC and BIC criteria. The model exhibiting the lowest AIC or BIC was selected to balance fit and complexity. By following this rigorous process, we ensured that only variables with substantial predictive power were retained in the final model.

4. You mentioned that the propensity score was estimated using logistic regression. It would be appropriate to list this model, at least in the Annex of the paper, along with the evaluation criteria of its performance.

**Response:** The propensity score was estimated using binary logistic regression. The model specification, including the list of covariates, is detailed in the Annex. To assess the model’s performance, we employed the Hosmer-Lemeshow goodness-of-fit test and calculated the area under the receiver operating characteristic (ROC) curve. Both measures indicated satisfactory model performance. Additionally, to enhance the propensity score model, variables significantly associated with earnings but not directly predictive of program participation (e.g., unemployment duration, father's occupation, job-related information) were incorporated. Detailed results, including model diagnostics, are available in the supplementary information.

5. In the Methodology section, you mentioned that the participants and non-participants were matched by propensity score matching. However, there is very little information about the matching itself. Did you use matching with or without replacement? How was the quality of the matching evaluated? How many units were in the samples after matching?

**Response**: Propensity score matching was employed to create comparable treatment and control groups. Nearest neighbor matching with replacement and a caliper width of 0.15 were utilized to ensure similar propensity scores between matched units. This process resulted in a balanced sample of 480 units, with 240 in each group.

Post-matching analysis demonstrated significantly improved covariate balance, as indicated by standardized differences approaching zero and variance ratios nearing one. Balance plots confirmed a substantial reduction in covariate imbalances. Additionally, the overlap of propensity score distributions between the treatment and control groups indicated common support, validating the matching process.

**Reply to Reviewer 2,**

1. The scientific gap that the authors are trying to fill is not defined in the Introduction. The main purpose of the article is also not identified.

**Response:** The Introduction has been revised to explicitly articulate the research gap and the study's primary objective. We have clearly outlined the existing knowledge in the field and identified the specific areas where our research contributes new insights. By doing so, we aim to enhance the reader's understanding of the study's significance and its contribution to the broader body of knowledge.

2. The literature review is out of date and does not provide a sufficient description of the current state of the art.

**Response:** The literature review has been substantially updated to incorporate recent studies and provide a comprehensive overview of the field's current state. By including the latest research findings, we aim to offer a more accurate and informative context for our study, strengthening its contribution to the existing body of knowledge.

3. The methods used are described only very briefly. It is not clear what matching technique the authors used. Given that most of these techniques are very data consuming, I don't consider the sample size to be sufficient.

**Response**: To address these concerns, we employed nearest neighbor matching with replacement and a caliper width of 0.15 for propensity score matching. This technique enabled us to create comparable treatment and control groups while effectively utilizing the available data. Although matching methods can be computationally intensive, our expanded dataset of 480 units (240 per group) provided sufficient power for the analysis. Post-matching assessments, including standardized differences, variance ratios, and balance plots, confirmed the achievement of adequate covariate balance.

4. Even the achieved results are described only very briefly, e.g. the description of the logistic regression model is missing and it is not clear whether the common support condition is met.

**Response:** We appreciate the reviewer's feedback. To address these concerns, we have expanded the methodology and results sections. The logistic regression model used to estimate propensity scores is now detailed, including the specific variables, coefficients, and model performance metrics (Hosmer-Lemeshow test, AUC). Furthermore, we have explicitly addressed the common support condition by demonstrating substantial overlap in the propensity score distributions for the treatment and control groups (Figure 4). These enhancements provide a more comprehensive understanding of our methodological approach and the validity of our findings.

5. In the discussion of the article, there is no comparison of the achieved results with the results of other authors.

**Response**: We appreciate the feedback and have taken steps to enhance the discussion section by providing comparisons of our achieved results with those of other authors, particularly focusing on impact analysis. By integrating these comparisons, we aim to contextualize our findings within the broader literature and highlight similarities or differences in outcomes across different studies. This addition enriches the discussion, offering readers a deeper understanding of the significance and implications of our results in relation to existing research.

6. The text of the article itself is often too divided into short paragraphs containing only one sentence. The tables are formatted inconsistently and the article lacks images that would illustrate the authors' findings and claims.

**Response**: To enhance readability and clarity, we've made several improvements to the article's structure and presentation. Paragraphs have been revised to improve flow and coherence, while tables have been formatted consistently with clear titles and headings. We've incorporated visual elements such as graphs, charts, and diagrams to illustrate key findings and support textual claims. Informative captions accompany all visuals to aid reader comprehension.

Once again, we would like to thank the editor and the reviewers for their constructive comments and suggestions.

        Sincerely yours,
