# Supplementary material for: Predictors of youth unemployment duration and impact evaluation of job creation program in East Gojjam Zone
Source: PLoS One. 2025 Apr 4;20(4):e0320795. doi: 10.1371/journal.pone.0320795 (PMC11970665; doi:10.1371/journal.pone.0320795)
Supplement: Attachment — Submitted filename: Response to Reviewers_2nd.docx [file pone.0320795.s007.docx]

Authors’ responses to the comments and suggestions from reviewers on the manuscript “Predictors of Youth Unemployment Duration and Impact Evaluation of Job Creation Program in East Gojjam Zone”

**Authors:** Nigusie Gashaye Shita ([nigusie27@gmail.com](mailto:nigusie27@gmail.com))

Metadel Azeze Mekonnen: mekonmeti@gmail.com

Yeshiwas Ewinetu Tegegne: yeshiwasewinetu@gmail.com

Misganaw Mekonnen Nigussie: misganaa2004@yahoo.com

Awoke Fetahi Woudneh: [fetahi.aw@gmail.com](mailto:fetahi.aw@gmail.com)

**Version:** 2 **Date:** October 31, 2024

**Author’s response to Editorial Board Member and reviews:**

Dear **Editorial Board Member and reviews**,

Thank you very much for your comments and suggestions to improve our manuscript.

Responses to some of the comments are addressed below, and the rest of the comments and suggestions are incorporated in the manuscript. Kindly note that changes are highlighted in yellow in the main manuscript.

**Reply to Reviewer 1.**

1. It is very unusual to use a significance level of 0.20. To be honest, I have never seen such a level in some scientific article. Why did you choose such a level?

**Response:** We selected a significance level of 0.20 for bivariate analyses and 0.10 for multivariate analyses to guide variable selection in the Cox regression and binary logistic models (1-3), while a p-value of less than 5% was considered significant for identifying associations in the final model.

This decision reflects the exploratory nature of our study, which aims to identify potential relationships and generate hypotheses rather than confirm existing theories. A higher significance level allows for a broader examination of variables, increasing the likelihood of uncovering significant associations.

Furthermore, using a higher significance level helps mitigate the risk of Type II errors, especially with smaller sample sizes, thereby enhancing statistical power. We also followed established guidelines, such as those from Hosmer and Lemeshow (1999), recommending significance levels between 20% and 25% during initial model selection.

Additionally, we employed a stepwise model-building process, incorporating both forward selection and backward elimination. This method allows for a thorough evaluation of variables and their interactions, ensuring that only those with meaningful contributions are retained in the final model.

2. Please correct the format of p-values in your tables and text. It is incorrect to write "0.000" because the value probably is not zero, although some software uses this incorrect format.

**Response**: We have corrected the formatting to ensure accuracy and clarity, eliminating the use of "0.000" and instead presenting p-values in a more precise manner.

3. If you used matching with replacement, how is it possible to have exactly the same number of participants and non-participants in your samples? It sounds very unlikely. Or I did not understand correctly whether 240 is the number of participants and non-participants before or after the matching procedure.

**Response:** To clarify, the figure of 240 refers to the number of observations in each group after matching. Initially, there were 240 total observations (96 participants and 144 non-participants). After applying matching with replacement, we achieved 240 matched observations in both the treated and control groups.

This method allows for equal numbers of participants and non-participants in the matched samples, despite initial differences in group sizes. While it may seem unusual, the goal is to balance the characteristics of both groups. For more details, please refer to S5Table.

4. The last paragraph on page 17 is repeated twice.

**Response:** We have amended the manuscript by removing the duplicated paragraph on page 17.

5. The discussion section lacks a comparison with more similar studies published so far. There is only one study mentioned for comparison. Be careful about the formatting of your paper. It is very different throughout the paper (for example, various font types).

**Response:** We have revised the formatting of the paper and enhanced the discussion to include comparisons with relevant studies where applicable. As noted in the introduction, there is a gap in research on predictors of unemployment duration. Consequently, our discussion emphasizes available evidence, including mean and median durations, as well as factors influencing unemployment likelihood. We highlight how certain factors can increase the likelihood of employment, indicating that shorter durations of unemployment can positively impact reemployment chances, while longer durations may reduce them.

**Reply to Reviewer 2.**

1. There is practically no literature review in the manuscript. The sources used in the introduction are still out of date.

**Response:** We have incorporated more up-to-date sources in the manuscript, focusing on relevant evidence. Additionally, we have provided an empirical literature review in the introduction section, as outlined in paragraphs 4 and 5, in accordance with journal formatting guidelines.

2. In the section devoted to methodology, it would be appropriate to state how (in what steps) the authors proceeded with their research.

**Response:** We have revised this section to include a detailed, step-by-step account of the research process, outlining the design, data collection, and analysis methods used.

3. The division of the manuscript is still relatively unclear. It would be more appropriate to use a structured division of headings and fewer headings overall.

**Response:** We have revised it to provide clearer organization, implementing a more structured division of headings and reducing the overall number of headings. However, in the methodology and results sections, we have retained different headings to emphasize specific points for the reader's clarity, in accordance with journal formatting guidelines.

4. I do not think that the formatting of the manuscript is followed, e.g., tables and a list of references. Also, some images are missing captions, e.g., individual axes, and e.g., there is an error in Figure 4.

**Response:** We had amended the format of the tables and had corrected an error in Figure 4. Additionally, we had ensured that all images had appropriate captions. The references had been cited automatically using EndNote, and we have reviewed them to ensure they meet the required Vancouver formatting standards, in accordance with the journal’s guidelines.

Once again, we would like to thank the editor and the reviewers for their constructive comments and suggestions.

Sincerely yours,

# Reference

 1.	Hosmer Jr D, Lemeshow S. Applied Survival Analysis [Análisis de Supervivencia Aplicado]. Nueva York: Wiley. 1999.

2.	Collett D. Modelling survival data in medical research: Chapman and Hall/CRC; 2023.

3.	Hosmer Jr DW, Lemeshow S, Sturdivant RX. Applied logistic regression: John Wiley & Sons; 2013.
