# Supplementary material for: Predictors of youth unemployment duration and impact evaluation of job creation program in East Gojjam Zone
Source: PLoS One. 2025 Apr 4;20(4):e0320795. doi: 10.1371/journal.pone.0320795 (PMC11970665; doi:10.1371/journal.pone.0320795)
Supplement: Attachment — Submitted filename: Response to Reviewers_3nd.docx [file pone.0320795.s008.docx]

Authors’ responses to the comments and suggestions from reviewers on the manuscript “Predictors of Youth Unemployment Duration and Impact Evaluation of Job Creation Program in East Gojjam Zone”

**Authors:** Nigusie Gashaye Shita ([nigusie27@gmail.com](mailto:nigusie27@gmail.com))

Metadel Azeze Mekonnen: mekonmeti@gmail.com

Yeshiwas Ewinetu Tegegne: yeshiwasewinetu@gmail.com

Misganaw Mekonnen Nigussie: misganaa2004@yahoo.com

Awoke Fetahi Woudneh: [fetahi.aw@gmail.com](mailto:fetahi.aw@gmail.com)

**Version:** 3 **Date:** December 08, 2024

**Author’s response to Editorial Board Member and reviews:**

Dear **Editorial Board Member and reviews**,

Thank you very much for your comments and suggestions to improve our manuscript.

Responses to some of the comments are addressed below, and the rest of the comments and suggestions are incorporated in the manuscript. Kindly note that changes are highlighted in yellow in the main manuscript.

**Reply to Reviewer 2.**

1. I still have one comment: the methodology is too structured with too many subtitles.

**Response**: We have revised the methodology by removing the subtitles for "Sample Size Determination" and "Inclusion and Exclusion Criteria," and incorporated these details into the "Study Population and Sampling Methods" section. Additionally, we have merged the "Study Variables" and "Operational Definitions" into one section. This revision reduces the number of subtitles and enhances the clarity of the methodology.

**Reply to Reviewer 3.**

1. The manuscript does not contain hypotheses or research questions. Formulating research questions would improve the study's focus. These should be stated at the end of the literature review along with the main research objectives.

**Response**: We have included the research questions at the end of the literature review, along with the main research objectives, in the introduction section, following the manuscript submission guidelines of *PLOS ONE*. This study addresses two key research questions: (1) what are the main factors influencing the duration of youth unemployment in East Gojjam? (2) How do job creation initiatives affect the duration and outcomes of youth unemployment in the region, including earnings?

2. The references in the manuscript are relevant and mostly recent, though updating a few foundational studies could incorporate additional trends in youth unemployment and job creation programs. The manuscript lacks a literature review, which should definitely be added before the methodology section.

**Response**: We have updated the manuscript by incorporating both recent and foundational studies to reflect current trends in youth unemployment and job creation programs. In line with PLOS ONE guidelines, we have added a structured literature review section before the methodology, within the introduction. Since the introduction already covers the background, key literature, relevant controversies, and study aims, we included the empirical literature review in paragraphs 3 and 4 to ensure compliance with the journal’s formatting requirements.

**Reply to Reviewer 4.**

1. Figures 1 and 5 need revision. In Figure 1, the frequency and percentage distribution should be displayed in separate columns. In Table 5, are the frequencies accurately presented? The numbers seem to indicate an extremely low response rate relative to the 240 respondents.

**Response**: We have revised Table 1 by separating the frequency and percentage distributions into distinct columns, as requested. However, in previous interactions, we maintained the current format, as it is common practice to display both frequency and percentage in a single column. As indicated in the table's column name, the first number represents the frequency, while the percentage is shown in parentheses. This format is widely used for clarity and conciseness when submitting manuscripts.

Regarding Table 5, the frequencies are accurate. The data is based on feedback from 30 key informants, including community leaders, local government officials, program coordinators, participants of job creation programs, and youth representatives, selected from six distinct districts to assess public perception of job creation programs. Three participants chose not to provide responses, which is why the table includes data from the 27 participants who provided responses, rather than all 240 respondents.

2. Most of the references in the manuscript are relevant, but it is essential to add at least 15-20 additional recent references, preferably indexed in Scopus and no older than five years, to strengthen the manuscript.

**Response**: To our knowledge, there is no specific limit on the number of references; however, we have made an effort to incorporate additional recent references, besides the reports, to strengthen the manuscript. These references, primarily sourced from Scopus-indexed journals, focus on the latest trends and research related to youth unemployment and job creation programs. This ensures the manuscript reflects the most current academic developments in the field.

**Reply to Reviewer 5.**

1. Opportunities for enhancement encompass the want for a more explicit rationale regarding the elevated importance criteria and a comprehensive examination of the program retention issues and their consequences. Broadening the discourse to juxtapose findings with analogous studies would enhance the contextualization of the results and fortify their significance.

**Response**: we have provided a clearer and more explicit rationale for the elevated importance criteria. Additionally, we have conducted a detailed examination of program retention issues and their implications, offering deeper insights into their consequences. To further enhance the contextualization of our results, we have included comparisons with analogous studies, thereby strengthening the significance and relevance of our findings.

Additionally, we have conducted a more comprehensive examination of program retention issues and their implications. To strengthen the contextualization of our findings, we have also included comparisons with analogous studies, which enhance the significance of our results

2. The statistical analysis has been performed with an adequate level of rigor and is well-suited to the study's objectives. The Cox regression appropriately models time-to-event data, and PSM effectively reduces selection bias in evaluating program impacts. The use of descriptive statistics, survival curves, and diagnostic tests for model validation further demonstrates methodological robustness. However, the higher-than-standard significance thresholds (p ≤ 0.20 for bivariate analyses) raise concerns about false positives, necessitating a stronger justification. Additionally, while balance diagnostics for PSM are mentioned, detailed reporting of covariate balance metrics (e.g., standardized mean differences) is missing. Addressing these gaps and including sensitivity analyses would enhance the transparency and reliability of the statistical findings.

**Response:** We employed a multi-step approach in our analysis. The 0.20 significance level was used during the exploratory phase to identify a broader range of potential relationships without prematurely narrowing the scope. This is consistent with standard practices in hypothesis generation. In subsequent steps, we applied more conservative significance thresholds: 0.10 for Cox regression and binary logistic regression, and 0.05 for the final models. These refined thresholds effectively mitigated the risk of false positives, as the initial 0.20 level was only used during exploratory analysis.

Regarding the balance diagnostics for PSM, we have included standardized mean differences and variance ratios for both raw and matched data in Supplementary Table S5. The standardized mean differences and variance ratios for the treatment and control groups tend toward zero and one, respectively, indicating that the PSM model is adequate for evaluating the impacts of the program. This suggests that the matching process was successful in reducing the selection bias between the groups. Besides, we have already included sensitivity analyses and show enhance the transparency and reliability of the statistical findings.

3. The authors state that all relevant data underlying the findings are included within the manuscript. They further indicate that ethical considerations, such as participant confidentiality and the presence of identifying information, prevent the provision of the complete raw dataset. However, they offer to share anonymized or aggregated data upon request to the corresponding author. While this demonstrates an effort to balance transparency with ethical obligations, fully aligning with open data standards would require more explicit details about how and under what conditions interested researchers can access the data. Providing a publicly available repository or a formal process for requesting access could enhance the credibility and reproducibility of the study.

**Response:** We are committed to transparency and will provide anonymized or aggregated data upon reasonable request. Access can be requested through a formal written inquiry to the corresponding author, ensuring compliance with ethical standards while enhancing the study’s credibility and reproducibility.

4. A certain places may require additional enhancement. Some sentences in the document are excessively long, potentially hindering readability, and there are minor discrepancies in formatting and grammar. For instance, problems with p-value formatting (e.g., the use of "0.000") and redundant content, such as the repeated paragraph on page 17, diminish the overall presentation. Furthermore, although the discourse is logical, it is devoid of comparisons to analogous studies, which would furnish a more comprehensive context for the results. Rectifying these flaws and conducting a comprehensive assessment of language and formatting would improve clarity, professionalism, and readability.

**Response:** We have revised excessively long sentences to improve readability, corrected formatting and grammatical discrepancies (including p-value formatting), and removed redundant content, such as the repeated paragraph on page 17, in prior revisions. Additionally, we have incorporated comparisons to analogous studies to provide greater context for our findings. A thorough review of language and formatting has been conducted to enhance clarity, professionalism, and readability.

Once again, we would like to thank the editor and the reviewers for their constructive comments and suggestions.

 Sincerely yours,

# Reference

 1.	Hosmer Jr D, Lemeshow S. Applied Survival Analysis [Análisis de Supervivencia Aplicado]. Nueva York: Wiley. 1999.

2.	Collett D. Modelling survival data in medical research: Chapman and Hall/CRC; 2023.

3.	Hosmer Jr DW, Lemeshow S, Sturdivant RX. Applied logistic regression: John Wiley & Sons; 2013.
