# Supplementary material for: Predictors of youth unemployment duration and impact evaluation of job creation program in East Gojjam Zone
Source: PLoS One. 2025 Apr 4;20(4):e0320795. doi: 10.1371/journal.pone.0320795 (PMC11970665; doi:10.1371/journal.pone.0320795)
Supplement: Attachment — Submitted filename: Review 2024.12.docx [file pone.0320795.s009.docx]

**Review**

The study addresses a timely and significant issue, particularly relevant to the context of developing countries. The research effectively employs Cox regression and propensity score matching (PSM) techniques, which are appropriately justified and applied to achieve the stated objectives.

The data collection process is meticulously planned, and the documentation of adherence to ethical principles is exemplary. Additionally, the study pays close attention to the role of various demographic and economic factors that influence the duration of unemployment.

Overall, the research is methodologically sound and contributes valuable insights into the dynamics of youth unemployment and the impact of job creation programs. However certain aspects could be improved to enhance its clarity, depth, and practical application.

**Areas for improvement in the study:**

1. Data limitations: The research considers only young people registered with the micro- and small enterprise office in the East Gojjam region. This significantly limits the generalizability of the results.
2. Interpretation of statistical results: The study does not show significant differences in monthly earnings between the treatment and control groups, yet it emphasizes the importance of job creation programs. This is contradictory and requires more detailed interpretation.
3. Lack of in-depth examination of program impacts: There is insufficient specific data on the weaknesses of job creation programs, such as the reasons for participant dissatisfaction. Detailing these aspects would increase the practical relevance of the research.
4. Limited literature context: While the study references some previous research, the lack of a broader literature review makes it unclear how the new findings contribute to the advancement of the topic. The number of referenced studies is very narrow and needs to be expanded. Furthermore, the exploration of causal relationships and the demonstration of critical thinking are missing.
5. Lack of comprehensive recommendations: Although the study makes recommendations for addressing the issues, these are insufficiently developed and not practically applicable.
6. Numerous grammatical and stylistic errors: These diminish the quality of the study. For instance

"The prevalence of employment was 33.3% (95% confidence interval: 27.3–39.3)." A more accurate phrasing would be: "The employment rate was 33.3% (95% confidence interval: 27.3–39.3)."

"Only 49% of the individuals who participated in the job creation initiatives expressed a desire to stay in the program." A better version could be: "Only 49% of individuals participating in the job creation initiatives expressed willingness to remain in the program."

Instead of "longer unemployed duration," the more precise term would be "longer duration of unemployment."

The references are inconsistent; for instance, some appear in parentheses, while others are placed at the end of sentences.

Incorrect word order:

*"The length of unemployment decreased by 0.27 months when the number of social networks among young people increased by one."* A more appropriate phrasing would be: *"An increase of one social network reduced unemployment duration by an average of 0.27 months."*

Missing transition words and cohesion: Certain parts of the text lack transitional words (e.g., *therefore, however, additionally*), which hinders the logical flow of the content.

**To summarise:**

The study addresses an important topic and is fundamentally well-structured; however, it has shortcomings in terms of the generalizability of the data, the interpretation of the results, and the level of detail in the recommendations.

With the suggested improvements, the research could significantly contribute to the development of policies aimed at reducing unemployment.
