# Supplementary material for: Predictors of youth unemployment duration and impact evaluation of job creation program in East Gojjam Zone
Source: PLoS One. 2025 Apr 4;20(4):e0320795. doi: 10.1371/journal.pone.0320795 (PMC11970665; doi:10.1371/journal.pone.0320795)
Supplement: Attachment — Submitted filename: Response to Reviewers_4nd.docx [file pone.0320795.s011.docx]

Authors’ responses to the comments and suggestions from reviewers on the manuscript “Predictors of Youth Unemployment Duration and Impact Evaluation of Job Creation Program in East Gojjam Zone”

**Authors:** Nigusie Gashaye Shita ([nigusie27@gmail.com](mailto:nigusie27@gmail.com))

Metadel Azeze Mekonnen: mekonmeti@gmail.com

Yeshiwas Ewinetu Tegegne: yeshiwasewinetu@gmail.com

Misganaw Mekonnen Nigussie: misganaa2004@yahoo.com

Awoke Fetahi Woudneh: [fetahi.aw@gmail.com](mailto:fetahi.aw@gmail.com)

**Version:** 4 **Date:** January 06, 2024

**Author’s response to Editorial Board Member and reviews:**

Dear **Editorial Board Member and reviews**,

Thank you very much for your comments and suggestions to improve our manuscript.

Responses to some of the comments are addressed below, and the rest of the comments and suggestions are incorporated in the manuscript. Kindly note that changes are highlighted in yellow in the main manuscript.

**Reply to Reviewer 6.**

1. Data limitations: The research considers only young people registered with the micro- and small enterprise office in the East Gojjam region. This significantly limits the generalizability of the results.

**Response:** The research focuses on young people registered with the micro- and small enterprise office in East Gojjam, which indeed limits the generalizability of the findings to the broader youth population. However, this focus provides valuable insights into the specific challenges and opportunities faced by young entrepreneurs in the formal sector, contributing to a deeper understanding of key barriers and enablers for addressing youth unemployment. This limitation has already been clearly acknowledged in the last paragraph of the Discussion section in Version 3 of the manuscript.

2. Interpretation of statistical results: The study does not show significant differences in monthly earnings between the treatment and control groups, yet it emphasizes the importance of job creation programs. This is contradictory and requires more detailed interpretation.

**Response:** The apparent contradiction has been addressed in the revised manuscript. Our analysis shows a statistically significant average treatment effect (ATE) of 1,069.7 birr, indicating that individuals in the treatment group earned, on average, 1,069.7 birr more per month compared to the control group. Additionally, the average treatment effect on the treated (ATET) was 1,285.1 birr, indicating that program participants earned an additional 1,285.1 birr per month. These results reinforce the positive impact of job creation programs on participants' earnings and clarify any misunderstandings in the interpretation of the findings.

3. Lack of in-depth examination of program impacts: There is insufficient specific data on the weaknesses of job creation programs, such as the reasons for participant dissatisfaction. Detailing these aspects would increase the practical relevance of the research.

**Response:** The study provides valuable insights into job creation programs but lacks a detailed examination of participant dissatisfaction. Our findings indicate that 20.8% of non-participants cited program incompatibility, and 37.5% identified governance issues as key reasons for non-engagement. Furthermore, the temporary nature of 61.5% of programs likely contributes to job insecurity and dissatisfaction. A supplementary survey conducted in East Gojjam revealed mixed satisfaction levels, with 63% of respondents viewing the programs as effective in promoting entrepreneurial motivation. However, concerns such as limited job access, governance challenges, and misalignment with participants' needs were also raised (see Table 5). These insights emphasize the need to address job stability, governance effectiveness, and alignment with participants' needs to improve satisfaction and enhance long-term program impact.

4. Limited literature context: While the study references some previous research, the lack of a broader literature review makes it unclear how the new findings contribute to the advancement of the topic. The number of referenced studies is very narrow and needs to be expanded. Furthermore, the exploration of causal relationships and the demonstration of critical thinking are missing.

**Response**: We have addressed the concern regarding the limited literature context by expanding the literature review in the introduction section, in line with the PLOS ONE submission format. The revised manuscript now includes a more comprehensive review of relevant studies, particularly in paragraphs 2, 3, and 4. We reviewed approximately 79 articles, providing a stronger foundation for the study and better contextualizing the findings. Additionally, we have made efforts to explore causal relationships and demonstrate critical thinking throughout the manuscript, ensuring that the study contributes meaningfully to advancing the topic. In the discussion section, we incorporated numerous recent studies indexed in well-known databases like Scopus, and we avoided citing non-indexed journals or articles older than five years, in accordance with previous reviewer comments.

To address your concern, we have summarized key studies on youth unemployment and job creation programs in Ethiopia. These studies examine the determinants of youth unemployment, identify methodological gaps, and assess the impact of job creation initiatives. By summarizing the following literature, we have incorporated the key points, along with other relevant studies, into the revised document.

**Related Literature Review**

The National Youth Policy of Ethiopia defines youth as individuals aged 15 to 29, which is the operational definition used in this study (Federal Negarit Gazette, 2019). According to the International Labour Organization (ILO), unemployment occurs when individuals in the labor force actively seeking work are unable to find employment at the prevailing market wage rate. Understanding the factors contributing to youth unemployment in Ethiopia is essential for achieving sustainable economic growth and poverty reduction. Additionally, job creation initiatives have varying impacts on resolving youth unemployment, depending on the type and design of the intervention.

A growing body of literature has explored the determinants of youth unemployment in Ethiopia, with many studies employing binary choice models and multivariate regression analysis. These studies typically treat youth unemployment as a binary outcome variable and examine various demographic, socio-economic, socio-cultural, and institutional factors as explanatory variables (Duguma & Tolcha, 2019; Mehari & Belay, 2017; Olkeba et al., 2023; Shuker et al., 2024; Tolesa & Zeleke, 2024; Hailie & Devi, 2024; Sileshi et al., 2024). Key findings indicate that youth unemployment is significantly influenced by factors such as regional disparities, sex, marital status, age, gender, education, limited access to job market information, financial constraints, lack of training, skill mismatches, lack of workplace opportunities, corruption, unfair competition, household size, and job search behavior.

Despite the valuable contributions of these studies, several methodological limitations persist. Many studies fail to address endogeneity issues arising from reverse causality or the omission of important variables. Furthermore, they do not use advanced econometric techniques, such as instrumental variable methods, fixed-effects models, or propensity score matching, which could mitigate biases and improve the accuracy of results. For example, Hailie & Devi (2024) rely on simple descriptive statistics, which prevent drawing valid inferences. Similarly, Shita & Dereje (2018) focus on urban youth unemployment in the East Gojjam Zone, Amhara Region, finding that age, work experience, skill match, social networks, and family prosperity negatively affect unemployment, while education and migration status positively influence it. However, the study is limited by its reliance on binary choice modeling, which offers limited precision in capturing the complexities of youth unemployment. Furthermore, there is a lack of empirical research that jointly examines youth unemployment duration and its welfare impacts, particularly in the context of Ethiopia.

To address these gaps, this study employed Cox regression to analyze the determinants of unemployment duration. Additionally, it used propensity score matching to evaluate the welfare effects of job creation initiatives, which help control for unobserved heterogeneity and sample selection bias.

Job creation programs in Ethiopia provide both immediate and long-term benefits by enhancing employment opportunities and improving income through various socio-economic factors. Programs that offer skills training and startup capital can empower youth to create businesses, addressing the root causes of unemployment (Berhe, 2021). Furthermore, promoting the transition of informal jobs to the formal sector can enhance job security and reduce unemployment rates (Yitateku, 2019). For instance, entrepreneurial programs have been shown to increase earnings by 33 percent and provide more stable working hours, while industrial jobs had no significant impact on employment or income after one year (Blattman & Dercon, 2018).

The Ethiopian government has made job creation a priority through social safety net programs such as the Productive Safety Net Program (PSNP) and the Urban Productive Safety Net and Jobs Project (UPSNJP), which focus on connecting disadvantaged workers to wage jobs (Maaskant et al., 2024). A light-touch job-facilitation intervention has been found to increase the likelihood of young female job seekers being employed, with higher earnings and savings; however, these impacts were short-lived (Vinez, 2024). Despite these efforts, many Ethiopians continue to experience stagnant incomes, as macroeconomic growth has not translated into significant improvements in earnings for the majority (Vinez, 2024). Moreover, the limited availability of wage employment opportunities and the adverse health impacts associated with factory work highlight the need for more comprehensive strategies to improve both job quality and income levels (Abebe et al., 2020).

While job creation programs have the potential to improve income, their effectiveness depends on addressing underlying economic barriers and improving the quality of available jobs. Furthermore, the outcomes of these programs vary across gender and geographic location. In light of these considerations, this study aimed to assess how job creation programs in the East Gojjam Zone have affected the income of youth beneficiaries compared to non-beneficiaries.

5. Lack of comprehensive recommendations: Although the study makes recommendations for addressing the issues, these are insufficiently developed and not practically applicable.

**Response**: We have addressed this concern by expanding and developing the recommendations to ensure they are more comprehensive and practically applicable. The revised recommendations now include detailed steps and strategies that can be implemented to address the issues identified in the study. These recommendations are designed to be actionable, considering the specific context of youth unemployment and job creation programs in Ethiopia.

6. Numerous grammatical and stylistic errors: These diminish the quality of the study.

**Response**: We have thoroughly addressed the grammatical and stylistic issues in version 3 of the manuscript. It seems that the updated document may not have reached you, as the necessary revisions have already been incorporated. Below is a summary of the specific changes made:

- **"The prevalence of employment was 33.3% (95% confidence interval: 27.3–39.3)."**
  Revised to: "The employment rate was 33.3% (95% confidence interval: 27.3–39.3)." This revision uses the more accurate term 'employment rate' rather than 'prevalence of employment.'
- **"Only 49% of the individuals who participated in the job creation initiatives expressed a desire to stay in the program."**

Revised to: "Only 49% of individuals participating in the job creation initiatives expressed a willingness to remain in the program." This revision clarifies that 49% of participants expressed interest in continuing the programs.

- **"Longer unemployed duration."**

Revised to: "Longer duration of unemployment," to improve precision in terminology.

- **Inconsistent references:**

We have already standardized the citation format throughout the manuscript, using the Vancouver reference style. The issue was not inconsistency in citation styles, but rather the placement of citations—either within the phrase or at the end of the sentence. This does not indicate inconsistency in citation styles, but a variation in citation placement. We have ensured uniform citation placement throughout the document.

- **"The length of unemployment decreased by 0.27 months when the number of social networks among young people increased by one."**

Revised to: "An increase of one social network reduced unemployment duration by an average of 0.27 months," which more accurately reflects the study's finding.

- **Missing transition words and cohesion:**

We have added the necessary transition words (e.g., therefore, however, additionally) to enhance the logical flow and overall coherence of the text.

**Response to Reviewer 7**

1. I can't see the literature section, I can't find the literature listed, this section definitely needs to be improved.

**Response**: We understand the importance of a clearly presented literature review. In accordance with PLOS ONE guidelines, we have structured the literature review within the introduction, placed before the methodology. The introduction covers the study’s background, key literature, relevant controversies, and aims. The empirical literature review is integrated into paragraphs 2, 3, and 4 to ensure a smooth flow and comply with the journal’s formatting requirements.

To address your concern, we have summarized key studies on youth unemployment and job creation programs in Ethiopia. These studies examine the determinants of youth unemployment, identify methodological gaps, and assess the impact of job creation initiatives. By summarizing the following literature, we have incorporated the key points, along with other relevant studies, into the revised document.

**Related Literature Review**

The National Youth Policy of Ethiopia defines youth as individuals aged 15 to 29, which is the operational definition used in this study (Federal Negarit Gazette, 2019). According to the International Labour Organization (ILO), unemployment occurs when individuals in the labor force actively seeking work are unable to find employment at the prevailing market wage rate. Understanding the factors contributing to youth unemployment in Ethiopia is essential for achieving sustainable economic growth and poverty reduction. Additionally, job creation initiatives have varying impacts on resolving youth unemployment, depending on the type and design of the intervention.

A growing body of literature has explored the determinants of youth unemployment in Ethiopia, with many studies employing binary choice models and multivariate regression analysis. These studies typically treat youth unemployment as a binary outcome variable and examine various demographic, socio-economic, socio-cultural, and institutional factors as explanatory variables (Duguma & Tolcha, 2019; Mehari & Belay, 2017; Olkeba et al., 2023; Shuker et al., 2024; Tolesa & Zeleke, 2024; Hailie & Devi, 2024; Sileshi et al., 2024). Key findings indicate that youth unemployment is significantly influenced by factors such as regional disparities, sex, marital status, age, gender, education, limited access to job market information, financial constraints, lack of training, skill mismatches, lack of workplace opportunities, corruption, unfair competition, household size, and job search behavior.

Despite the valuable contributions of these studies, several methodological limitations persist. Many studies fail to address endogeneity issues arising from reverse causality or the omission of important variables. Furthermore, they do not use advanced econometric techniques, such as instrumental variable methods, fixed-effects models, or propensity score matching, which could mitigate biases and improve the accuracy of results. For example, Hailie & Devi (2024) rely on simple descriptive statistics, which prevent drawing valid inferences. Similarly, Shita & Dereje (2018) focus on urban youth unemployment in the East Gojjam Zone, Amhara Region, finding that age, work experience, skill match, social networks, and family prosperity negatively affect unemployment, while education and migration status positively influence it. However, the study is limited by its reliance on binary choice modeling, which offers limited precision in capturing the complexities of youth unemployment. Furthermore, there is a lack of empirical research that jointly examines youth unemployment duration and its welfare impacts, particularly in the context of Ethiopia.

To address these gaps, this study employed Cox regression to analyze the determinants of unemployment duration. Additionally, it used propensity score matching to evaluate the welfare effects of job creation initiatives, which help control for unobserved heterogeneity and sample selection bias.

Job creation programs in Ethiopia provide both immediate and long-term benefits by enhancing employment opportunities and improving income through various socio-economic factors. Programs that offer skills training and startup capital can empower youth to create businesses, addressing the root causes of unemployment (Berhe, 2021). Furthermore, promoting the transition of informal jobs to the formal sector can enhance job security and reduce unemployment rates (Yitateku, 2019). For instance, entrepreneurial programs have been shown to increase earnings by 33 percent and provide more stable working hours, while industrial jobs had no significant impact on employment or income after one year (Blattman & Dercon, 2018).

The Ethiopian government has made job creation a priority through social safety net programs such as the Productive Safety Net Program (PSNP) and the Urban Productive Safety Net and Jobs Project (UPSNJP), which focus on connecting disadvantaged workers to wage jobs (Maaskant et al., 2024). A light-touch job-facilitation intervention has been found to increase the likelihood of young female job seekers being employed, with higher earnings and savings; however, these impacts were short-lived (Vinez, 2024). Despite these efforts, many Ethiopians continue to experience stagnant incomes, as macroeconomic growth has not translated into significant improvements in earnings for the majority (Vinez, 2024). Moreover, the limited availability of wage employment opportunities and the adverse health impacts associated with factory work highlight the need for more comprehensive strategies to improve both job quality and income levels (Abebe et al., 2020).

While job creation programs have the potential to improve income, their effectiveness depends on addressing underlying economic barriers and improving the quality of available jobs. Furthermore, the outcomes of these programs vary across gender and geographic location. In light of these considerations, this study aimed to assess how job creation programs in the East Gojjam Zone have affected the income of youth beneficiaries compared to non-beneficiaries.

**Reply to Reviewer 8.**

1. The study focuses on the factors that are essential in a given region to help job creation programmers have an impact. The methodology used is fully adapted to the research questions identified. Their results are relevant based on the research methods and their analysis and will help the region to address the problems effectively and strengthen the support system. The research highlights the problem of youth unemployment in parts of Ethiopia. Their research also highlights job creation initiatives, skills gaps, regional disparities and limited access to food. The study not only articulates the causes but also proposes solutions to achieve this goal. They identify all areas that can help and support inclusive growth through job creation based on their research. The researchers have revised their study accordingly, with improvements made to the research methodology, stylistic factors and structure. These revisions made the study fully fit for publication.

**Response**: We appreciate your thorough evaluation of our study and your recognition of its focus, methodological rigor, and relevance in addressing youth unemployment in Ethiopia. Your acknowledgment of the study’s contributions, particularly in identifying skills gaps, regional disparities, and job creation initiatives, reinforces its significance. We are pleased that the revisions to the methodology, structure, and presentation have enhanced the study's quality and suitability for publication.

Once again, we would like to thank the editor and the reviewers for their constructive comments and suggestions.

 Sincerely yours,

**References**

Abebe, G., Buehren, N., & Goldstein, M. (2020). Short-Run Welfare Impacts of Factory Jobs: Experimental Evidence from Ethiopia. World Bank Group, Africa Region, Gender Innovation Lab. July.

Berhe, M. W. (2021). Empirical Analysis of Urban Youth Unemployment in Ethiopia. African Development Review, 33(1), 104–116. <https://doi.org/10.1111/1467-8268.12514>

Blattman, C., & Dercon, S. (2018). The Impacts of Industrial and Entrepreneurial Work on Income and Health: Experimental Evidence from Ethiopia. American Economic Journal: Applied Economics, 10(3), 1–38. <https://doi.org/10.1257/app.20170173>

Duguma, A. L., & Tolcha, F. T. (2019). Determinants of Urban Youth Unemployment: The Case of Guder Town, Western Shoa Zone, Ethiopia. International Journal of Research -GRANTHAALAYAH, 7(8), 318–327. <https://doi.org/10.29121/granthaalayah.v7.i8.2019.674>

Gazette, F. N. (2019). Federal Negarit Gazette.

Hailie, Y. G., & Devi, M. U. (2024). Factors and Motives of Unemployed Youths Not to Engage in Own Business. 19(1), 301–305.

Mehari, A. T., & Belay, C. F. (2017). Challenges and Prospects of Entrepreneurship Development and Job Creation for Youth Unemployed: Evidence from Addis Ababa and Dire Dawa City Administrations, Ethiopia. <https://doi.org/10.1186/s13731-017-0070-3>

Olkeba, A., Dula, T., Gutema, P., Tolossa, D., Bekele, A. A., Olkeba, A., Dula, T., Gutema, P., Tolossa, D., & Mekuriaw, A. (2023). The Effects of Schooling on Rural Unemployment in Ethiopia. Cogent Economics & Finance, 11(2). <https://doi.org/10.1080/23322039.2023.2273599>

Shita, A., & Dereje, M. (2018). Determinants of Urban Youth Unemployment: Evidence from East Gojjam Zone, Ethiopia. International Journal of Economic Development, 11(2), 245–265.

Shuker, F. M., Sadik, H. H., Shuker, F. M., & Sadik, H. H. (2024). A Critical Review of Rural Youth Unemployment in Ethiopia. International Journal of Adolescence and Youth, 29(1). <https://doi.org/10.1080/02673843.2024.2322564>

Tolesa, S. F., & Zeleke, G. F. (2024). Analysis of Factors Affecting Unemployment: A Systematic Literature Review of Evidence from Ethiopia. Trends in Agricultural Sciences, 3(2), 91–99. <https://doi.org/10.17311/tas.2024.91.99>

Vinez, E. W. C., & M. (2024). Working Today for a Better Tomorrow in Ethiopia: Jobs for Poor and Vulnerable Households. <https://doi.org/10.1596/978-1-4648-2020-5>

Yitateku, A. (2019). Determinants of Rural Youth Unemployment in Yilmanadensa District, West Gojjam Zone, Amhara Region, Ethiopia.
